# Time on drug analysis based on real life data

**DOI:** 10.7448/IAS.17.4.19790

**Published:** 2014-11-02

**Authors:** Eugen Schülter, Rolf Kaiser, Maurizio Zazzi, Anders Sönnerborg, Ricardo Camacho, Jens Verheyen

**Affiliations:** 1 Institute of Virology, University of Cologne, Cologne, Germany; 2 Department of Microbiology, University of Siena, Siena, Italy; 3 Clinical Microbiology and Infectious Disease, Karolinska Institute, Stockholm, Sweden; 4 Laboratory of Molecular Biology, Hospital Egas Moniz, Lisbon, Portugal; 5 Institute of Virology, University of Duisburg-Essen, Essen, Germany

## Abstract

**Introduction:**

The health condition of HIV-1 infected patients has improved during the last years, but lifelong antiretroviral treatment is still needed. However resistance, multiple side effects and drug to drug interactions of antiretrovirals challenge the establishment of a long lasting regimen. The average running time of each antiretroviral drug composing the therapy episodes combination antiretroviral therapy (cART) may be seen as an indicator of effectiveness and tolerability.

**Materials and Methods:**

To evaluate the running time of each drug used in HIV-1 treatment, we extracted therapy episodes from the latest release of the EuResist database (www.euresist.org). The evaluation period was from Oct 2006 to Oct 2012. Inclusion criteria for this analysis were continuous patient monitoring for at least two years (i.e. latest therapy start in Oct 2010), and the extraction of at least 100 cases per drug analyzed. Drug intake interruptions of less than a month were ignored.

**Results:**

At the time of data extraction (Feb 2013), the EuResist database contained data from 61,953 patients of which 11,499 fulfilled the inclusion criteria. We obtained 37,035 drug treatment lines from 38,153 cARTs and the overall average length of drug intake was 18.7 months. For each single drug these average durations measured in months were: 18.3 (3TC); 20.8 (ABC); 12.3 (d4T); 14.3 (ddI); 23.2 (FTC); 23.0 (TDF); 13.4 (ZDV); 19.8 (EFV); 21.9 (ETR); 17.7 (NVP); 19.2 (ATV); 22.7 (DRV); 18.7 (FPV); 17.9 (LPV); 15.2 (SQV); 14.6 (TPV); 22.6 (RAL); 21.9 (MVC) and 8.9 (T20). Overall drug discontinuation rates at one, two and three years were 35.0, 48.8 and 95.8%, respectively. Average discontinuation rates for the different drug classes at two years these were: 46.2% for NRTIs; 49.7% for NNRTIs; 55.4% for PIs and 37.6% for Raltegravir/Maraviroc.

**Conclusions:**

In this cohort the overall frequency of therapy changes is high. After two years of treatment, on average 49% of the patients change at least one drug in their cART. Thus, we have to expect numerous changes in the long term perspective of treatments. The observed differences in durations suggest that newer drugs might have advantages over older ones. However possible reasons and confounding factors (such as number of past treatment lines, co-medication, risk group, etc.) were not addressed at this time of the analysis.

